# The genome sequence of the Eurasian Spoonbill, *Platalea leucorodia* Linnaeus, 1758 (Pelecaniformes: Threskiornithidae)

**DOI:** 10.12688/wellcomeopenres.24883.1

**Published:** 2025-09-15

**Authors:** Michelle F. O’Brien, Rosa Lopez Colom, Natural History Museum Genome Acquisition Lab, Wellcome Sanger Institute Tree of Life Management, Samples and Laboratory team, Wellcome Sanger Institute Scientific Operations: Sequencing Operations, Wellcome Sanger Institute Tree of Life Core Informatics team, Tree of Life Core Informatics collective, Darwin Tree of Life Consortium

**Affiliations:** 1 Wildfowl & Wetlands Trust, Slimbridge, Gloucestershire, England, UK

**Keywords:** Platalea leucorodia; Eurasian Spoonbill; genome sequence; chromosomal; Pelecaniformes

## Abstract

We present a genome assembly from an individual female *Platalea leucorodia* (Eurasian Spoonbill; Chordata; Aves; Pelecaniformes; Threskiornithidae). The assembly contains two haplotypes with total lengths of 1 345.83 megabases and 1 190.44 megabases. Most of haplotype 1 (95.57%) is scaffolded into 37 chromosomal pseudomolecules, including the W and Z sex chromosomes. Haplotype 2 was assembled to scaffold level. The mitochondrial genome has also been assembled, with a length of 17.15 kilobases. This assembly was generated as part of the Darwin Tree of Life project, which produces reference genomes for eukaryotic species found in Britain and Ireland.

## Species taxonomy

Eukaryota; Opisthokonta; Metazoa; Eumetazoa; Bilateria; Deuterostomia; Chordata; Craniata; Vertebrata; Gnathostomata; Teleostomi; Euteleostomi; Sarcopterygii; Dipnotetrapodomorpha; Tetrapoda; Amniota; Sauropsida; Sauria; Archelosauria; Archosauria; Dinosauria; Saurischia; Theropoda; Coelurosauria; Aves; Neognathae; Neoaves; Aequornithes; Pelecaniformes; Threskiornithidae; *Platalea*; *Platalea leucorodia* Linnaeus, 1758 (NCBI:txid257867)

## Background

The Eurasian Spoonbill ( *Platalea leucorodia*) is a tall white bird, with a white feather crest on the head and a yellow area at the base of the neck seen in breeding adults. They stand 70–95 cm tall, with a weight of 1130–1960 g and wingspan of 115–135 cm. Males are larger than females in this species and have longer bills and legs ( Matheu *et al.*, 2020). These birds reach sexual maturity at 3 to 4 years of age and the oldest ringed bird was reported to be 28 years of age ( Matheu *et al.*, 2020).

This species inhabits shallow, large wetlands which include rivers, marshes, larger lakes and flooded areas. It can also be found in estuarine habitats, tidal creaks and lagoons (particularly during the winter months). Nesting takes place on islands in lakes or rivers and nests are made in dense plant matter such as reedbeds, bushes or trees ( Matheu *et al.*, 2020). Nests are often colonial and usually 1-2m apart. There are usually 3 or 4 eggs in a clutch and incubation can be up to 25 days with fledging taking place after 45 to 50 days ( Cramp & Simmons, 1977).

Their diet consists of invertebrates, amphibians and fish and occasional algae or plant material. Foraging takes place as the bird wades through water (often in small flocks) and sweeps the bill from side to side. The significance of bill shape has a number of hypotheses including potential reduction in turbulence ( Swennen & Yu, 2008) or generating suction ( Weihs & Katzir, 2008).

Distribution of the Eurasian spoonbill is relatively complex, with the species breeding across Eurasia and wintering in North Africa, sub-Saharan Africa and Eurasia ( Xi *et al.*, 2021). There are currently three subspecies that have been identified: a West African subspecies ( *Platalea leucorodia balsaci*), which breeds mainly in Mauritania and islands along the coast ( Piersma *et al.*, 2012); a Red Sea subspecies ( *P. l. archeri*), which breeds in the Red Sea and along the coast of Somalia; and the Eurasian Spoonbill ( *P.l. leucorodia*) which has a wide distribution throughout Eurasia. This third group is the nominate subspecies and consists of four biogeographical populations currently ( Wetlands International, 2021). First, a West Europe/West Mediterranean and West Africa population, which breeds along the western coast of Europe and winters in the western Mediterranean and the western coast of Africa (numbers are estimated at 11 300 birds) ( Champagnon *et al.*, 2019); second, a Southeast European/Mediterranean and tropical African population, wintering in the Mediterranean or tropical areas of northern Africa (numbers are estimated at 11 700 birds) ( Champagnon *et al.*, 2019); third, a West/Southwest and South Asian population, which breeds across Central and Southwest Asia and winters in Southwest and South Asia (numbers are estimated at 23 000 birds) ( Wetlands International, 2021); and finally, an East Asian population, breeding mostly in Northeast Asia, Mongolia and China, and wintering in Southeast China, South Korea and Japan, (numbers are estimated at 20 000 birds) ( Xi *et al.*, 2021).

Analysis of the genetic differences between subspecies and the genetic differentiation between geographic populations is particularly important in this species to ensure that subspecies are correctly recorded ( Piersma *et al.*, 2012). Hybridisation has also been recorded between Eurasian and Black-faced spoonbills ( Matheu *et al.*, 2020).

The main threats to this species include habitat loss and degradation, overfishing and disturbance, poaching and collision with overhead power lines ( del Hoyo *et al.*, 1992; Hancock *et al.*, 1992; Triplet *et al.*, 2008).

**Figure 1.**
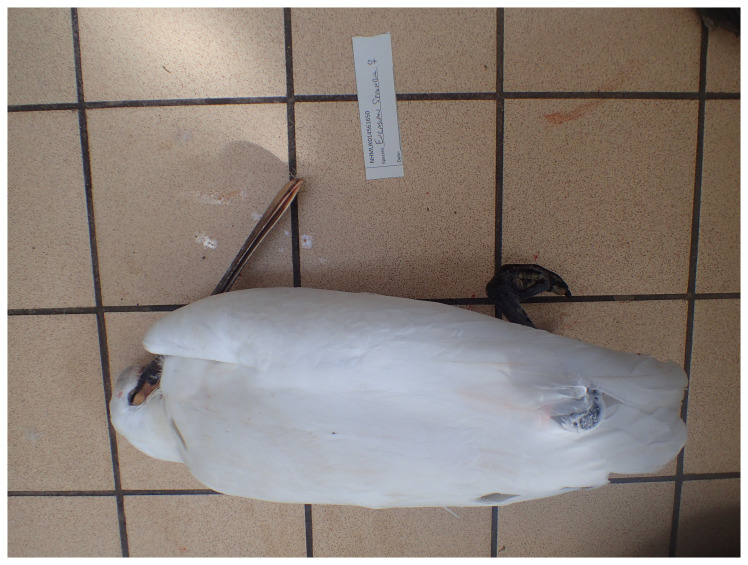
Photograph of the *Platalea leucorodia* (bPlaLeu2) specimen used for genome sequencing.

This species is listed as Least Concern on the IUCN Red List both globally and in Europe ( BirdLife International, 2019; BirdLife International, 2021). This is due to its extensive range, stable trend in population numbers and relative abundance. We present a chromosomally complete genome sequence for *Platalea leucorodia*, the Eurasian Spoonbill. The assembly was produced using the Tree of Life pipeline from a specimen collected in Newgounds, Gloucester, England, United Kingdom ( Figure 1), as part of the Darwin Tree of Life project.

## Methods

### Sample acquisition

The specimen used for genome sequencing was an adult female *Platalea leucorodia* (specimen ID NHMUK014561650, ToLID bPlaLeu2; Figure 1), collected from Newgounds, Gloucester, England, United Kingdom (latitude 51.73, longitude –2.4) on 2022-02-02. This was a captive specimen found dead. The specimen was collected and identified by Michelle O’Brien (Wildfowl & Wetlands Trust). Several small samples of pectoral muscle were taken and stored at –80 °C. A sample from the same specimen was used for RNA sequencing. Sample metadata were collected in line with the Darwin Tree of Life project standards described by Lawniczak *et al.* (2022).

### Nucleic acid extraction

Protocols for high molecular weight (HMW) DNA extraction developed at the Wellcome Sanger Institute (WSI) Tree of Life Core Laboratory are available on protocols.io ( Howard *et al.*, 2025). The bPlaLeu2 sample was weighed and triaged to determine the appropriate extraction protocol. Tissue from the muscle was homogenised by cryogenic disruption using the Covaris cryoPREP ^®^ Automated Dry Pulverizer.

HMW DNA was extracted in the WSI Scientific Operations core using the Automated MagAttract v2 protocol. DNA was sheared into an average fragment size of 12–20 kb following the Megaruptor®3 for LI PacBio protocol. Sheared DNA was purified by manual SPRI (solid-phase reversible immobilisation). The concentration of the sheared and purified DNA was assessed using a Nanodrop spectrophotometer and Qubit Fluorometer using the Qubit dsDNA High Sensitivity Assay kit. Fragment size distribution was evaluated by running the sample on the FemtoPulse system. For this sample, the final post-shearing DNA had a Qubit concentration of 15.37 ng/μL and a yield of 691.65 ng, with a fragment size of 11.8 kb. The 260/280 spectrophotometric ratio was 1.8, and the 260/230 ratio was 2.27.

RNA was extracted from muscle tissue of bPlaLeu2 in the Tree of Life Laboratory at the WSI using the RNA Extraction: Automated MagMax™ *mir*Vana protocol. The RNA concentration was assessed using a Nanodrop spectrophotometer and a Qubit Fluorometer using the Qubit RNA Broad-Range Assay kit. Analysis of the integrity of the RNA was done using the Agilent RNA 6000 Pico Kit and Eukaryotic Total RNA assay.

### PacBio HiFi library preparation and sequencing

Library preparation and sequencing were performed at the WSI Scientific Operations core. Libraries were prepared using the SMRTbell Prep Kit 3.0 (Pacific Biosciences, California, USA), following the manufacturer’s instructions. The kit includes reagents for end repair/A-tailing, adapter ligation, post-ligation SMRTbell bead clean-up, and nuclease treatment. Size selection and clean-up were performed using diluted AMPure PB beads (Pacific Biosciences). DNA concentration was quantified using a Qubit Fluorometer v4.0 (ThermoFisher Scientific) and the Qubit 1X dsDNA HS assay kit. Final library fragment size was assessed with the Agilent Femto Pulse Automated Pulsed Field CE Instrument (Agilent Technologies) using the gDNA 55 kb BAC analysis kit.

The sample was sequenced using the Sequel IIe system (Pacific Biosciences, California, USA). The concentration of the library loaded onto the Sequel IIe was in the range 40–135 pM. The SMRT link software, a PacBio web-based end-to-end workflow manager, was used to set-up and monitor the run, and to perform primary and secondary analysis of the data upon completion.

### Hi-C

***Sample preparation and crosslinking***

The Hi-C sample was prepared from 20–50 mg of frozen muscle tissue of the bPlaLeu2 sample using the Arima-HiC v2 kit (Arima Genomics). Following the manufacturer’s instructions, tissue was fixed and DNA crosslinked using TC buffer to a final formaldehyde concentration of 2%. The tissue was homogenised using the Diagnocine Power Masher-II. Crosslinked DNA was digested with a restriction enzyme master mix, biotinylated, and ligated. Clean-up was performed with SPRISelect beads before library preparation. DNA concentration was measured with the Qubit Fluorometer (Thermo Fisher Scientific) and Qubit HS Assay Kit. The biotinylation percentage was estimated using the Arima-HiC v2 QC beads.

***Hi-C library preparation and sequencing***

Biotinylated DNA constructs were fragmented using a Covaris E220 sonicator and size selected to 400–600 bp using SPRISelect beads. DNA was enriched with Arima-HiC v2 kit Enrichment beads. End repair, A-tailing, and adapter ligation were carried out with the NEBNext Ultra II DNA Library Prep Kit (New England Biolabs), following a modified protocol where library preparation occurs while DNA remains bound to the Enrichment beads. Library amplification was performed using KAPA HiFi HotStart mix and a custom Unique Dual Index (UDI) barcode set (Integrated DNA Technologies). Depending on sample concentration and biotinylation percentage determined at the crosslinking stage, libraries were amplified with 10–16 PCR cycles. Post-PCR clean-up was performed with SPRISelect beads. Libraries were quantified using the AccuClear Ultra High Sensitivity dsDNA Standards Assay Kit (Biotium) and a FLUOstar Omega plate reader (BMG Labtech).

Prior to sequencing, libraries were normalised to 10 ng/μL. Normalised libraries were quantified again and equimolar and/or weighted 2.8 nM pools. Pool concentrations were checked using the Agilent 4200 TapeStation (Agilent) with High Sensitivity D500 reagents before sequencing. Sequencing was performed using paired-end 150 bp reads on the Illumina NovaSeq 6000.

### RNA library preparation and sequencing

Libraries were prepared using the NEBNext ^®^ Ultra™ II Directional RNA Library Prep Kit for Illumina (New England Biolabs), following the manufacturer’s instructions. Poly(A) mRNA in the total RNA solution was isolated using oligo(dT) beads, converted to cDNA, and uniquely indexed; 14 PCR cycles were performed. Libraries were size-selected to produce fragments between 100–300 bp. Libraries were quantified, normalised, pooled to a final concentration of 2.8 nM, and diluted to 150 pM for loading. Sequencing was carried out on the Illumina NovaSeq 6000 to generate 150-bp paired-end reads.

### Genome assembly

Prior to assembly of the PacBio HiFi reads, a database of *k*-mer counts ( *k* = 31) was generated from the filtered reads using FastK. GenomeScope2 ( Ranallo-Benavidez *et al.*, 2020) was used to analyse the *k*-mer frequency distributions, providing estimates of genome size, heterozygosity, and repeat content.

The HiFi reads were assembled using Hifiasm in Hi-C phasing mode ( Cheng *et al.*, 2021; Cheng *et al.*, 2022), producing two haplotypes. Hi-C reads ( Rao *et al.*, 2014) were mapped to the primary contigs using bwa-mem2 ( Vasimuddin *et al.*, 2019). Contigs were further scaffolded with Hi-C data in YaHS ( Zhou *et al.*, 2023), using the --break option for handling potential misassemblies. The scaffolded assemblies were evaluated using Gfastats ( Formenti *et al.*, 2022), BUSCO ( Manni *et al.*, 2021) and MERQURY.FK ( Rhie *et al.*, 2020).

The mitochondrial genome was assembled using MitoHiFi ( Uliano-Silva *et al.*, 2023), which runs MitoFinder ( Allio *et al.*, 2020) and uses these annotations to select the final mitochondrial contig and to ensure the general quality of the sequence.

### Assembly curation

The assembly was decontaminated using the Assembly Screen for Cobionts and Contaminants ( ASCC) pipeline. TreeVal was used to generate the flat files and maps for use in curation. MicroFinder ( Mathers *et al.*, 2025) was used to order scaffolds prior to curation. Manual curation was conducted primarily in PretextView and HiGlass ( Kerpedjiev *et al.*, 2018). Scaffolds were visually inspected and corrected as described by Howe *et al.* (2021). Manual corrections included 53 breaks, 235 joins, and removal of 9 haplotypic duplications. The curation process is documented at https://gitlab.com/wtsi-grit/rapid-curation. PretextSnapshot was used to generate a Hi-C contact map of the final assembly.

### Assembly quality assessment

The Merqury.FK tool ( Rhie *et al.*, 2020) was run in a Singularity container ( Kurtzer *et al.*, 2017) to evaluate *k*-mer completeness and assembly quality for both haplotypes using the *k*-mer databases ( *k* = 31) computed prior to genome assembly. The analysis outputs included assembly QV scores and completeness statistics.

The genome was analysed using the BlobToolKit pipeline, a Nextflow implementation of the earlier Snakemake version ( Challis *et al.*, 2020). The pipeline aligns PacBio reads using minimap2 ( Li, 2018) and SAMtools ( Danecek *et al.*, 2021) to generate coverage tracks. It runs BUSCO ( Manni *et al.*, 2021) using lineages identified from the NCBI Taxonomy ( Schoch *et al.*, 2020). For the three domain-level lineages, BUSCO genes are aligned to the UniProt Reference Proteomes database ( Bateman *et al.*, 2023) using DIAMOND blastp ( Buchfink *et al.*, 2021). The genome is divided into chunks based on the density of BUSCO genes from the closest taxonomic lineage, and each chunk is aligned to the UniProt Reference Proteomes database with DIAMOND blastx. Sequences without hits are chunked using seqtk and aligned to the NT database with blastn ( Altschul *et al.*, 1990). The BlobToolKit suite consolidates all outputs into a blobdir for visualisation. The BlobToolKit pipeline was developed using nf-core tooling ( Ewels *et al.*, 2020) and MultiQC ( Ewels *et al.*, 2016), with containerisation through Docker ( Merkel, 2014) and Singularity ( Kurtzer *et al.*, 2017).

## Genome sequence report

### Sequence data

**Figure 2.**
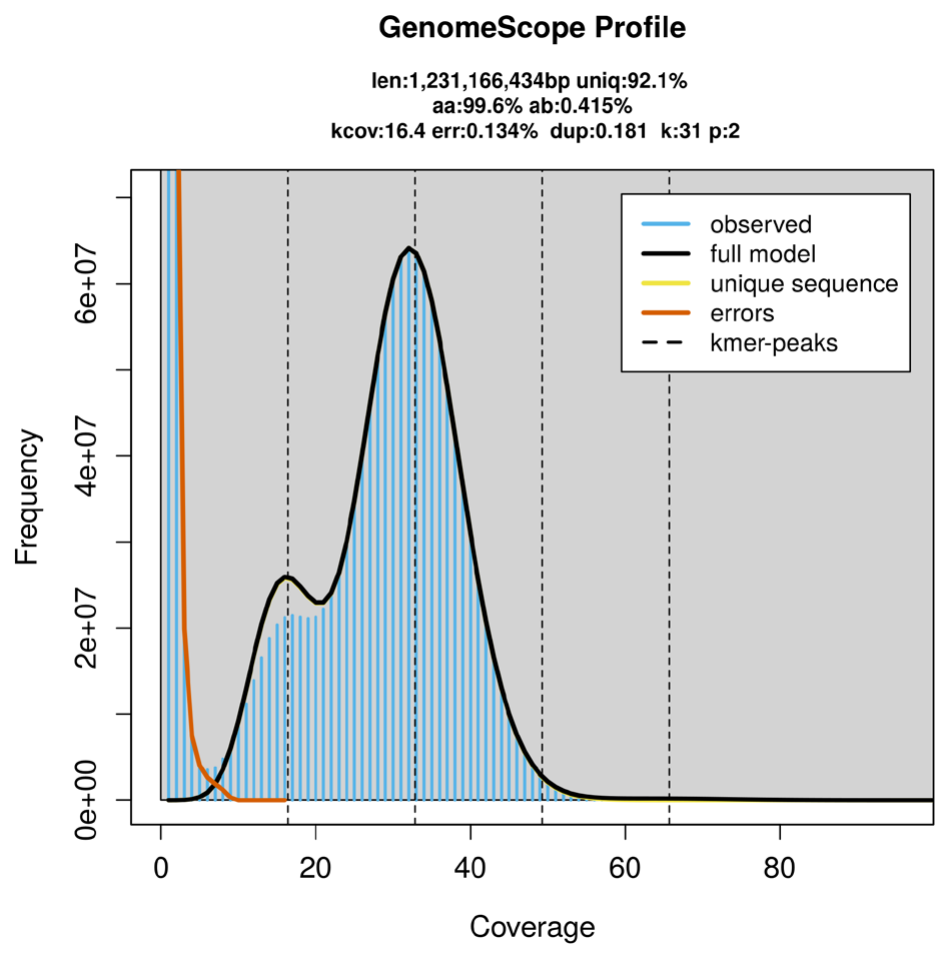
Frequency distribution of *k*-mers generated using GenomeScope2. The plot shows observed and modelled *k*-mer spectra, providing estimates of genome size, heterozygosity, and repeat content based on unassembled sequencing reads.

PacBio sequencing of the *Platalea leucorodia* specimen generated 42.31 Gb (gigabases) from 4.85 million reads, which were used to assemble the genome. GenomeScope2.0 analysis estimated the haploid genome size at 1 231.17 Mb, with a heterozygosity of 0.41% and repeat content of 7.96% ( Figure 2). These estimates guided expectations for the assembly. Based on the estimated genome size, the sequencing data provided approximately 33× coverage. Hi-C sequencing produced 77.64 Gb from 514.17 million reads, which were used to scaffold the assembly. RNA sequencing data were also generated and are available in public sequence repositories. Table 1 summarises the specimen and sequencing details.

**Table 1.** Specimen and sequencing data for BioProject PRJEB70851.

Platform	PacBio HiFi	Hi-C	RNA-seq
**ToLID**	bPlaLeu2	bPlaLeu2	bPlaLeu2
**Specimen ID**	NHMUK014561650	NHMUK014561650	NHMUK014561650
**BioSample (source individual)**	SAMEA112468120	SAMEA112468120	SAMEA112468120
**BioSample (tissue)**	SAMEA112468133	SAMEA112468133	SAMEA112468133
**Tissue**	muscle	muscle	muscle
**Instrument**	Sequel IIe	Illumina NovaSeq 6000	Illumina NovaSeq 6000
**Run accessions**	ERR12353021; ERR12353022	ERR12356293	ERR12356294
**Read count total**	4.85 million	514.17 million	61.18 million
**Base count total**	42.31 Gb	77.64 Gb	9.24 Gb

### Assembly statistics

The genome was assembled into two haplotypes using Hi-C phasing. Haplotype 1 was curated to chromosome level, while haplotype 2 was assembled to scaffold level. The final assembly has a total length of 1 345.83 Mb in 668 scaffolds, with 703 gaps, and a scaffold N50 of 94.71 Mb ( Table 2).

**Table 2.** Genome assembly statistics.

**Assembly name**	bPlaLeu2.hap1.1	bPlaLeu2.hap2.1
**Assembly accession**	GCA_965183815.1	GCA_965183785.1
**Assembly level**	chromosome	scaffold
**Span (Mb)**	1 345.83	1 190.44
**Number of chromosomes**	37	N/A
**Number of contigs**	1 371	1 012
**Contig N50**	3.3 Mb	3.6 Mb
**Number of scaffolds**	668	408
**Scaffold N50**	94.71 Mb	102.18 Mb
**Longest scaffold length (Mb)**	169.23	N/A
**Sex chromosomes**	W and Z	N/A
**Organelles**	Mitochondrion: 17.15 kb	N/A

**Figure 3.**
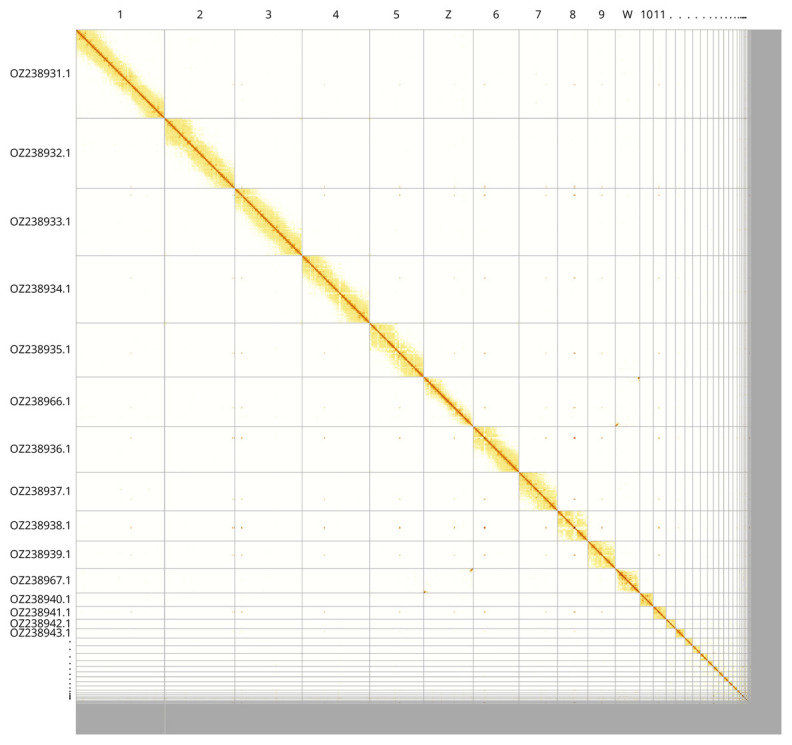
Hi-C contact map of the *Platalea leucorodia* genome assembly. Assembled chromosomes are shown in order of size and labelled along the axes, with a megabase scale shown below. The plot was generated using PretextSnapshot.

Most of the assembly sequence (95.57%) was assigned to 37 chromosomal-level scaffolds, representing 35 autosomes and the W and Z sex chromosomes. These chromosome-level scaffolds, confirmed by Hi-C data, are named according to size ( Figure 3; Table 3). Curation of this assembly resulted in 35 autosomes, plus Z and W. The published karyotype is ± 70 ( Takagi & Sasaki, 1974).

**Table 3.** Chromosomal pseudomolecules in the haplotype 1 genome assembly of *Platalea leucorodia* bPlaLeu2.

INSDC accession	Molecule	Length (Mb)	GC%
OZ238931.1	1	169.66	41
OZ238932.1	2	133.58	41
OZ238933.1	3	128.51	41.50
OZ238934.1	4	128.45	41.50
OZ238935.1	5	103.04	42.50
OZ238936.1	6	87.21	41
OZ238937.1	7	73.81	42.50
OZ238938.1	8	57.75	43.50
OZ238939.1	9	52.14	44
OZ238940.1	10	25.39	45
OZ238941.1	11	24.62	46
OZ238942.1	12	17.91	46.50
OZ238943.1	13	17.68	46.50
OZ238944.1	14	14.84	47.50
OZ238945.1	15	14.57	49
OZ238946.1	16	14.10	47.50
OZ238947.1	17	10.99	48.50
OZ238948.1	18	10.22	51.50
OZ238949.1	19	9.57	53
OZ238950.1	20	9.13	50
OZ238951.1	21	8.08	54.50
OZ238952.1	22	8.07	53
OZ238953.1	23	5.15	58
OZ238954.1	24	3.81	59
OZ238955.1	25	2.64	51
OZ238956.1	26	2.37	62.50
OZ238957.1	27	2.32	54.50
OZ238958.1	28	1.74	61.50
OZ238959.1	29	1.69	62.50
OZ238960.1	30	1.34	53.50
OZ238961.1	31	1.17	54
OZ238962.1	32	0.92	60.50
OZ238963.1	33	0.72	64.50
OZ238964.1	34	0.62	59
OZ238965.1	35	0.52	62.50
OZ238967.1	W	47.12	44
OZ238966.1	Z	94.71	41.50

The mitochondrial genome was also assembled. This sequence is included as a contig in the multifasta file of the genome submission and as a standalone record.

**Figure 4.**
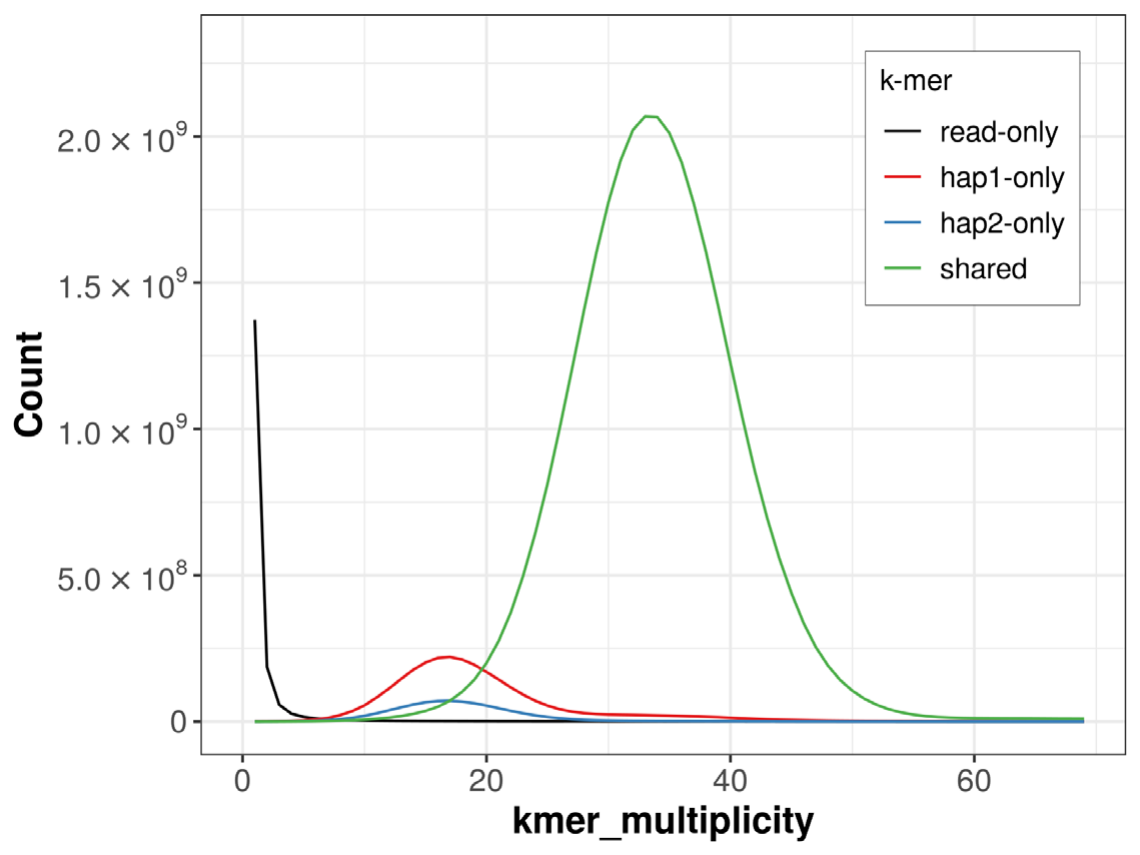
Evaluation of *k*-mer completeness using MerquryFK. This plot illustrates the recovery of *k*-mers from the original read data in the final assemblies. The horizontal axis represents *k*-mer multiplicity, and the vertical axis shows the number of *k*-mers. The black curve represents *k*-mers that appear in the reads but are not assembled. The green curve corresponds to *k*-mers shared by both haplotypes, and the red and blue curves show *k*-mers found only in one of the haplotypes.

For haplotype 1, the estimated QV is 62.1, and for haplotype 2, 62.8. When the two haplotypes are combined, the assembly achieves an estimated QV of 62.4. The *k*-mer completeness is 95.62% for haplotype 1, 86.85% for haplotype 2, and 99.61% for the combined haplotypes ( Figure 4).

**Figure 5.**
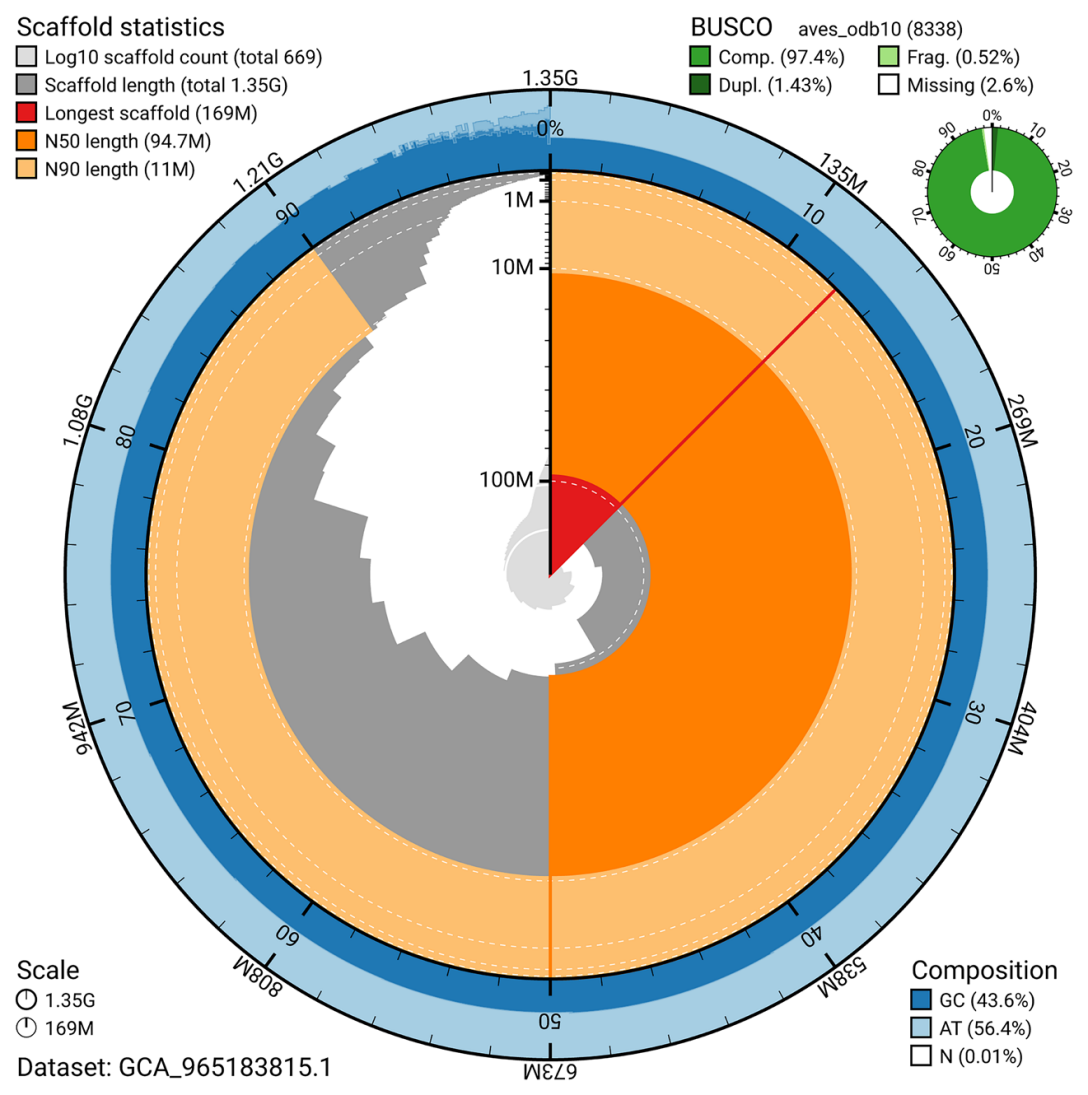
Assembly metrics for bPlaLeu2.hap1.1. The BlobToolKit snail plot provides an overview of assembly metrics and BUSCO gene completeness. The circumference represents the length of the whole genome sequence, and the main plot is divided into 1 000 bins around the circumference. The outermost blue tracks display the distribution of GC, AT, and N percentages across the bins. Scaffolds are arranged clockwise from longest to shortest and are depicted in dark grey. The longest scaffold is indicated by the red arc, and the deeper orange and pale orange arcs represent the N50 and N90 lengths. A light grey spiral at the centre shows the cumulative scaffold count on a logarithmic scale. A summary of complete, fragmented, duplicated, and missing BUSCO genes in the set is presented at the top right. An interactive version of this figure can be accessed on the BlobToolKit viewer.

**Figure 6.**
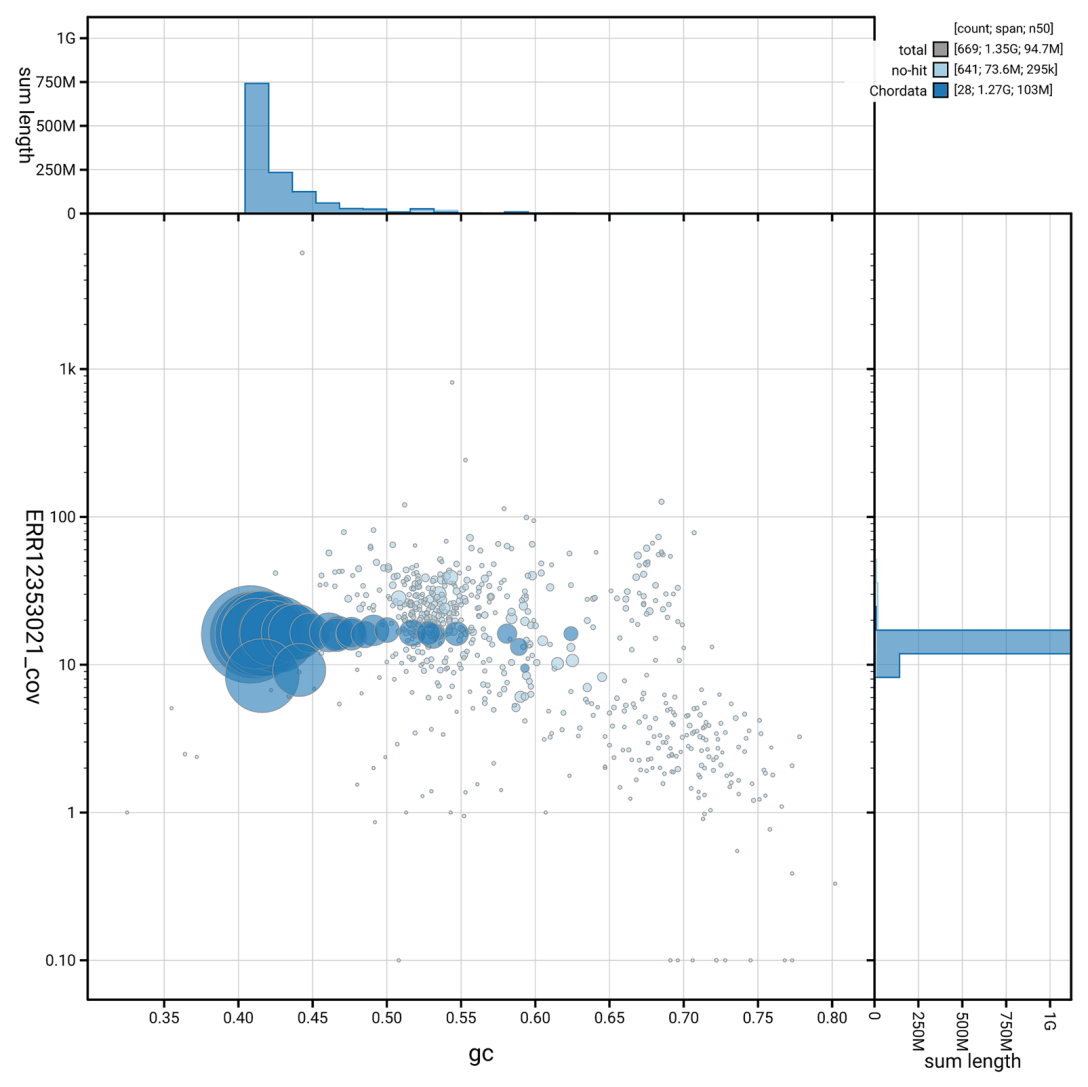
BlobToolKit GC-coverage plot for bPlaLeu2.hap1.1. Blob plot showing sequence coverage (vertical axis) and GC content (horizontal axis). The circles represent scaffolds, with the size proportional to scaffold length and the colour representing phylum membership. The histograms along the axes display the total length of sequences distributed across different levels of coverage and GC content. An interactive version of this figure is available on the BlobToolKit viewer.

BUSCO analysis using the aves_odb10 reference set ( *n* = 8 338) identified 97.4% of the expected gene set (single = 96.0%, duplicated = 1.4%) for haplotype 1. The snail plot in Figure 5 summarises the scaffold length distribution and other assembly statistics for haplotype 1. The blob plot in Figure 6 shows the distribution of scaffolds by GC proportion and coverage for haplotype 1.

Table 4 lists the assembly metric benchmarks adapted from Rhie *et al.* (2021) the Earth BioGenome Project Report on Assembly Standards September 2024. The EBP metric, calculated for the haplotype 1, is **6.C.Q62**, meeting the recommended reference standard.

**Table 4.** Earth Biogenome Project summary metrics for the *Platalea leucorodia* assembly.

Measure	Value	Benchmark
EBP summary (haplotype 1)	6.C.Q62	6.C.Q40
Contig N50 length	3.30 Mb	≥ 1 Mb
Scaffold N50 length	94.71 Mb	= chromosome N50
Consensus quality (QV)	Haplotype 1: 62.1; haplotype 2: 62.8; combined: 62.4	≥ 40
*k*-mer completeness	Haplotype 1: 95.62%; Haplotype 2: 86.85%; combined: 99.61%	≥ 95%
BUSCO	C:97.4% [S:96.0%; D:1.4%]; F:0.5%; M:2.1%; n:8338	S > 90%; D < 5%
Percentage of assembly assigned to chromosomes	95.57%	≥ 90%

### Wellcome Sanger Institute – Legal and Governance

The materials that have contributed to this genome note have been supplied by a Darwin Tree of Life Partner. The submission of materials by a Darwin Tree of Life Partner is subject to the **‘Darwin Tree of Life Project Sampling Code of Practice’**, which can be found in full on the Darwin Tree of Life website. By agreeing with and signing up to the Sampling Code of Practice, the Darwin Tree of Life Partner agrees they will meet the legal and ethical requirements and standards set out within this document in respect of all samples acquired for, and supplied to, the Darwin Tree of Life Project. Further, the Wellcome Sanger Institute employs a process whereby due diligence is carried out proportionate to the nature of the materials themselves, and the circumstances under which they have been/are to be collected and provided for use. The purpose of this is to address and mitigate any potential legal and/or ethical implications of receipt and use of the materials as part of the research project, and to ensure that in doing so we align with best practice wherever possible. The overarching areas of consideration are:

    •    Ethical review of provenance and sourcing of the material

    •    Legality of collection, transfer and use (national and international)

Each transfer of samples is further undertaken according to a Research Collaboration Agreement or Material Transfer Agreement entered into by the Darwin Tree of Life Partner, Genome Research Limited (operating as the Wellcome Sanger Institute), and in some circumstances, other Darwin Tree of Life collaborators.

## Data Availability

European Nucleotide Archive: Platalea leucorodia (Eurasian spoonbill). Accession number PRJEB70851. The genome sequence is released openly for reuse. The *Platalea leucorodia* genome sequencing initiative is part of the Darwin Tree of Life Project (PRJEB40665), the Sanger Institute Tree of Life Programme (PRJEB43745) and Vertebrate Genomes Project (PRJNA489243). All raw sequence data and the assembly have been deposited in INSDC databases. The genome will be annotated using available RNA-Seq data and presented through the Ensembl pipeline at the European Bioinformatics Institute. Raw data and assembly accession identifiers are reported in Table 1 and Table 2. Production code used in genome assembly at the WSI Tree of Life is available at https://github.com/sanger-tol. Table 5 lists software versions used in this study.

## Author information

Contributors are listed at the following links:

- Members of the Natural History Museum Genome Acquisition Lab
- Members of the Wellcome Sanger Institute Tree of Life Management, Samples and Laboratory team
- Members of Wellcome Sanger Institute Scientific Operations – Sequencing Operations
- Members of the Wellcome Sanger Institute Tree of Life Core Informatics team
- Members of the Tree of Life Core Informatics collective
- Members of the Darwin Tree of Life Consortium
