# Supplementary material for: The genome sequence of the Eurasian Spoonbill, Platalea leucorodia Linnaeus, 1758 (Pelecaniformes: Threskiornithidae)
Source: Wellcome Open Res. 2025 Sep 15;10:511. [Version 1] doi: 10.12688/wellcomeopenres.24883.1 (PMC12529062; doi:10.12688/wellcomeopenres.24883.1)
Supplement: Peer review history

# Reviewer report: Reviewer response for version 1

**DOI:** 10.21956/wellcomeopenres.27408.r133385

**Published:** 2025-10-25

**Authors:** Bengt Hansson

**Affiliations:** 1 Lund University, Lund, Sweden

The manuscript presents a high-quality genome assembly of *Platalea leucorodia* (Eurasian Spoonbill), generated as part of the Darwin Tree of Life project. The authors report two haplotypes with substantial coverage and chromosomal scaffolding, including the W and Z sex chromosomes, as well as a complete mitochondrial genome. The methods appear sound and appropriate for the scope of the project, and the results are clearly presented and well contextualized.

 I am in general happy with the content, the methodological approach, and the presentation of the results. The assembly represents a valuable contribution to avian genomics and biodiversity research.

 One minor point: the photo in Figure 1 appears to be upside down and should be corrected.

 I recommend the manuscript for indexing with no major revisions.

Are sufficient details of methods and materials provided to allow replication by others?

Yes

Is the rationale for creating the dataset(s) clearly described?

Yes

Are the datasets clearly presented in a useable and accessible format?

Yes

Are the protocols appropriate and is the work technically sound?

Yes

Reviewer Expertise:

Evolutionary biology, population genetics, sex chromosome evolution, avian genomics

I confirm that I have read this submission and believe that I have an appropriate level of expertise to confirm that it is of an acceptable scientific standard.

# Reviewer report: Reviewer response for version 1

**DOI:** 10.21956/wellcomeopenres.27408.r133386

**Published:** 2025-10-15

**Authors:** Ricardo Jorge Lopes

**Affiliations:** 1 CE3C - Centre for Ecology, Evolution and Environmental Changes & CHANGE - Global Change and Sustainability Institute, Departamento de Biologia, Faculdade de Ciências, Universidade de Lisboa, Lisboa, Portugal

This paper describes the procedures and results of a reference genome assembly of a female adult European spoonbill ( *Platalea leucorodia*). I find the methodology robust, and well documented and the genome assembly of high quality. It is the second genome for the Genus (There is a genome of *Platalea minor* - black-faced spoonbill), and it would be pertinent to say this and provide some comparative summary, especially if you find significant differences.

 I have some minor comments:

**1. Sample acquisition**

 - Concerning the voucher, the bird was found dead, but the cause of death is not mentioned. I think it is important to inform if the death was a random incident, or caused by a pathogen of other infirmity.  Was a necropsy made? If yes, was there any confirmation of cause of death?

 - Do you have information about the captivity status? Was the bird born in captivity? If so, please inform about the age. If not, please give some information about the origin of the bird.

**2. Assembly curation**

 Did the ASCC pipeline identified any cobiont? If so, please report them. Does the submitted Run accessions files are real raw files or the files after going through this pipeline? 

**3. Data availability section**

 Should the mention of Platalea leucorodia be in italic? Please check.

**4. Figure 6**

 BlobToolKit GC-coverage plot - Design wise, the legend position, as it is, the break of the border of the plot is not "nice".

Are sufficient details of methods and materials provided to allow replication by others?

Yes

Is the rationale for creating the dataset(s) clearly described?

Yes

Are the datasets clearly presented in a useable and accessible format?

Yes

Are the protocols appropriate and is the work technically sound?

Partly

Reviewer Expertise:

I work mainly with Conservation Genetics, actively promoting the production of high quality reference genomes.

I confirm that I have read this submission and believe that I have an appropriate level of expertise to confirm that it is of an acceptable scientific standard.

# Reviewer report: Reviewer response for version 1

**DOI:** 10.21956/wellcomeopenres.27408.r133388

**Published:** 2025-10-06

**Authors:** Subir Shakya

**Affiliations:** 1 Harvard University, Cambridge, Massachusetts, USA; 2 Department of Evolutionary Biology, LMU Munich, Planegg-Martinsreid, Bavaria, Germany

Background: If you would like a description of the bird, then perhaps including the spoon shaped bill is a must compared to the other traits described.

 Also, some of the background paragraphs are one line long. Perhaps coalesce a few of them into coherent paragraphs.

 Most of the methods and results are pretty standard and I do not have issues regarding any of them. One thing I may like to see is a circos plot comparing the scaffolds to the other ibis genome, since in the ibis there is a neo-sex chromosome and thus a comparison would help visualize if the neo-sex chromosome is shared in the entire family or if it is species specific.

Are sufficient details of methods and materials provided to allow replication by others?

Yes

Is the rationale for creating the dataset(s) clearly described?

Yes

Are the datasets clearly presented in a useable and accessible format?

Yes

Are the protocols appropriate and is the work technically sound?

Yes

Reviewer Expertise:

Comparative genomics

I confirm that I have read this submission and believe that I have an appropriate level of expertise to confirm that it is of an acceptable scientific standard.
